# In Vitro and In Vivo Anti-*Clostridioides difficile* Effect of a Probiotic *Bacillus amyloliquefaciens* Strain

**DOI:** 10.4014/jmb.2107.07057

**Published:** 2021-10-14

**Authors:** Md Imtiazul Islam, Hoonhee Seo, Asma Redwan, Sukyung Kim, Saebim Lee, Mashuk Siddiquee, Ho-Yeon Song

**Affiliations:** 1 Department of Microbiology and Immunology, School of Medicine, Soonchunhyang University, Cheonan 31151, Republic of Korea; 2 Probiotics Microbiome Convergence Center, Soonchunhyang University, Asan 31538, Republic of Korea

**Keywords:** *Clostridioides difficile*, *Bacillus amyloliquefaciens*, BA PMC-80, probiotic, antibiotic

## Abstract

*Clostridioides difficile* infection (CDI) is a significant cause of hospital-acquired and antibiotic-mediated intestinal diseases and is a growing global public health concern. Overuse of antibiotics and their effect on normal intestinal flora has increased the incidence and severity of infections. Thus, the development of new, effective, and safe treatment options is a high priority. Here, we report a new probiotic strain, *Bacillus amyloliquefaciens* (BA PMC-80), and its in vitro/in vivo anti-*C. difficile* effect as a prospective novel candidate for replacing conventional antibiotics. BA PMC-80 showed a significant anti-*C. difficile* effect in coculture assay, and its cell-free supernatant (CFS) also exhibited a considerable anti-*C. difficile* effect with an 89.06 μg/ml 50% minimal inhibitory concentration (MIC) in broth microdilution assay. The CFS was stable and equally functional under different pHs, heat, and proteinase treatments. It also exhibited a high sensitivity against current antibiotics and no toxicity in subchronic toxicity testing in hamsters. Finally, BA PMC-80 showed a moderate effect in a hamster CDI model with reduced infection severity and delayed death. However, further studies are required to optimize the treatment condition of the hamster CDI model for better efficacy and identify the antimicrobial compound produced by BA PMC-80.

## Introduction

*Clostridioides difficile*-mediated infection (CDI) is a common nosocomial digestive infection characterized by pseudomembranous colitis and mainly associated with antibiotic treatments [1]. *C. difficile* (CD) remains prevalent throughout North America and Europe [2, 3]. Approximately half a million cases of CDI and more than 29,000 associated deaths were reported annually in the United States, and related overall medical costs exceeded US$ 4 billion [4]. Reduction of CDI rates is still one of the top priorities of the Centers for Disease Control and Prevention (CDC) [5]. Several current studies report that CDI is emerging in Asia and other parts of the world, where its occurrence was believed to be low [6, 7]. Most commonly, CDI arises among people on antibiotic treatment, as antibiotics disrupt the normal gut microbiota, decreasing resistance to colonization and leaving the body vulnerable to this opportunistic pathogen. Major classes of antibiotics have been linked to the risk of CDI and a patient may remain susceptible to infection for up to 3 months post antibiotic cessation [8].

Recurrence of infection is one of the significant drawbacks of the current treatment options for CDI, such as vancomycin, metronidazole, and fidaxomicin [9,10]. Several alternative therapeutic options include immune-based therapies, fecal microbiota transplantation (FMT), and vaccinations [11]. Slight effectiveness of immune-based therapies has been found in clinical trials, as well as intravenous immunoglobulin (IVIG) [12] against severe CDI and human monoclonal antibodies against recurrent CDI [13, 14]. Although FMT is effective against refractory and recurrent CDI, the method is hard to standardize and has been linked to the risk of transmitting other infectious diseases [15-17]. The CDI-targeted vaccines are still in clinical trials [18]; however, their prolonged seroconversion time [18-21] makes them unsuited for providing rapid protection. Alternative treatment options are required and they must be effective against both primary and recurrent CDI while helping to restore the complex balance of the normal gut microbiota without disrupting the indigenous microbiota [22]. Probiotics are attractive as a potential alternative to standard antibiotic therapy as they can prevent the invasion of pathogens by repopulating beneficial microbiota and producing antimicrobial compounds [23]. Different probiotics such as *Lactobacillus rhamnosus* GG and *Saccharomyces boulardii* have already been reported to prevent antibiotic-associated and CDI-induced diarrhea [24-26]. Bacteria of the genus *Bacillus* are also well known for producing many antimicrobial peptides with different chemical structures, such as bacteriocins, bacteriocin-like substances, and lipopeptides [27-29]. *Bacillus amyloliquefaciens* (BA), a potential candidate and member of the *Bacillus* genus, has been suggested in several recent studies to produce antimicrobial peptides [27], antibiotic compounds such as difficidin and bacilysin [30, 31], antifungal protein baciamin [32, 33] and antifungal iturin/fengycin-like peptides [34]. BA is also known to exert a beneficial effect on CDI-associated diarrheal diseases [35].

Korean fermented foods are a recognized source of many probiotic bacteria [36, 37]. We therefore started a project focused on isolating and applying probiotics as new agents to replace conventional antibiotics in the fight against CDI. Initially, several prospective probiotic strains of various origins were tested against CD, and as a result, *B. amyloliquefaciens* (BA PMC-80) and its significant anti-CD effect were discovered. Here, we report the results of in vitro/in vivo efficacy and safety studies on the potential of BA PMC-80 for CDI therapy.

## Materials and Methods

### Commercial Drugs and Chemicals

Methicillin, streptomycin, rifampicin, vancomycin, and clindamycin were purchased from Sigma-Aldrich (USA).

### Bacterial Strain

*Clostridioides difficile* (ATCC 43255) with *tcdA* (Toxin A) and *tcdB* (Toxin B) was purchased from the American Type Culture Collection (USA).

### Isolation of Probiotic Strains from Korean Traditional Fermented Foods

Probiotic strains were isolated from traditional fermented foods such as abalone, sea conch, ghee, cured cheese, cured kimchi, kimchi (fresh), kimchi (old), mixed soybean sauce, mustard pickles, pepper sauce, radish kimchi, seaweed sauce, sesame leaf kimchi, soybean liquid, string cheese, white kimchi, and young radish kimchi from various regions of Korea. The liquid portion of each food sample was streaked on brain heart infusion agar (211065, BD Difco, USA), M17 agar (MB-M1192, Kisanbio, Korea), Tos-MUP agar (MB-T0892, Kisanbio, Korea), and de Man, Rogosa and Sharpe (MRS) agar (288210, BD difco, USA) media using a loop, respectively, and samples were then cultured in an aerobic (general incubator, N-Biotek, Korea) or anaerobic (Concept 400, Baker Ruskin Technologies, UK) incubator at 37°C, respectively.

### Screening for Potential Bacteria with Anti-CD Activity

Initially, anti-CD activity was investigated using agar diffusion assay according to Karsha-Wysocki *et al*. [38] with slight modifications. Briefly, overnight precultures of isolated probiotic bacteria were prepared from frozen glycerol stocks in MRS broth at 37°C in aerobic conditions. The next day, 3-μl probiotic precultures were spotted on the surface of MRS agar plates and incubated for 24 h at 37°C in aerobic condition to develop the growth of probiotic bacteria in the spot. After developing the probiotic bacterial spots, they were overlaid with 10 ml of BHI soft agar (0.7% agar) containing a 200-μl inoculum of an overnight culture of toxigenic *Clostridioides difficile* (ATCC 43255) in BHI broth. The plates were incubated for another 24 h under anaerobic condition at 37°C in an anaerobic chamber (Concept M 400, Baker Ruskinn, Canada).

### Identification of Selected Potential Strain

Biochemical and molecular identification techniques were used to identify PMC-80. According to the manufacturer’s instructions, the carbon source utilization profile of PMC-80 was determined to evaluate its biochemical characteristics using an API 50 CHB system (bioMerieux, Inc., France) [39].

Molecular identification of PMC-80 was carried out using partial 16S rRNA gene sequence analysis using two universal primers 518 F (forward primer) 5’-CCAGCAGCCGCGGTAATACG-3’ [40] and 805R (reverse primer) 5’-GACTACHVGGGTATCTAATCC-3’ [41]. PCR products were separated by agarose gel electrophoresis (1% w/v) and visualized by staining with ethidium bromide. Sequencings were performed using a Big Dye Terminator Cycle Sequencing Kit (Applied BioSystems, USA), and the sequencing products were resolved on an Applied Biosystems model 3730XL automated DNA sequencing system (Applied BioSystems, USA). The resulting sequences of PMC-80 were collected in FASTA format for further analysis.

### Phylogenetic Analysis

Similarities between the 16S rRNA gene sequence of PMC-80 and other strains in the National Center for Biotechnology Information (NCBI) database were evaluated in the Basic Local Alignment Search Tool (BLAST) program on the NCBI website (https://blast.ncbi.nlm.nih.gov/Blast.cgi). Then, the 16S rRNA gene sequences representing the highest hits from the BLAST were retrieved and aligned in MEGA-X software. A phylogenetic tree was built with the constructed multiple sequence alignment using the neighbor-joining method in MEGA-X software. PMC-80 was later renamed BA PMC-80 after identification.

### Evaluation of Anti-CD Activity of Cell-Free Supernatants (CFSs) of BA PMC-80

Cell-free supernatant (CFS) was used to determine the anti-CD activity of compounds secreted by BA PMC-80. CFS of BA PMC-80 was prepared according to Lee *et al*. [42] with slight modifications. Briefly, overnight culture of the BA PMC-80 incubated at 37°C in MRS broth (BD) was centrifuged at 10,000 ×g for 10 min at 4°C, followed by filtration through a 0.2-μm sterile filter (Millipore, USA). The filtrate (20 ml) was further transferred to a 3K MWCO Amicon Filter (Millipore, France) and centrifuged at 5,000 ×g until the volume was concentrated to 2 ml. The protein concentration was measured using the Pierce BCA Protein Assay Kit (Thermo Fisher Scientific, USA).

The MIC of the BA PMC-80 CFS was determined using standard broth microdilution assay [42], CFU enumeration assay, and luminescent microbial cell viability assay [43] with some modifications.

### Broth Microdilution Assay

In 96-well plates, *C. difficile* (ATCC 43255) inocula were prepared in BHI broth (BD, USA) to a final density of 5×10^5^ CFU/ml in 100 μl with 10 μl of two-fold serial diluted CFS and vancomycin and incubated overnight (18 h) in anaerobic condition (Concept M 400, Baker Ruskin, Canada) at 37°C. The final concentrations of CFS and vancomycin were 5700-2.78 μg/ml and 128-0.0625 μg/ml, respectively. Without any drug, the same concentration of bacterial cells with MRS broth was used as a positive control, and without any bacteria or drugs, only fresh media were used as the negative control. The ODs were measured every two hours at 600 nm with a multilabel reader (Victor X3, PerkinElmer, USA). After incubation, the concentration that reduced the 50% OD compared to the positive control was selected as the 50% minimum inhibitory concentration (MIC_50_).

To determine the number of viable CD cells after the CFS treatment, we performed the CFU enumeration assay using the CFS-treated CD cell suspension. Then, 10 μl of overnight incubated CFS-treated CD cell suspension was plated on a BHI agar plate after serial dilution. The plate was then incubated for a further 24 h at 37°C, and CD colonies were counted.

According to the manufacturer’s instructions, the viability of CFS-treated CD cells was also evaluated by measuring ATP production using a luminescence-based cell viability assay kit (Promega, USA). After overnight incubation, 50 μl of CFS-treated CD cells was collected and thoroughly resuspended in 50 μl of freshly prepared BacTiter-Glo reagent and incubated at room temperature for 10 min on an orbital shaker. Following incubation, luminescence was measured with a multilabel reader (Victor X3, PerkinElmer, USA).

### Evaluation of the Effect of Enzymatic Degradation, Heat, and pH on BA PMC-80 Activity

We evaluated the stability of BA PMC-80 CFS under different pHs, temperatures, and enzymatic degradation according to the protocol [39] with slight modifications. The anti-CD activity of the 1X MIC_50_ (89.06 μg/ml) concentration of BA PMC-80 CFS was determined in acidic (pH 5), neutral (pH 7) and basic (pH 9) conditions using broth microdilution assay as described in the previous section. To further evaluate the proteolytic stability and thermostability, CFS (1X MIC_50_) was treated with 2 mg/ml Proteinase K (Promega, USA) for 2 h at 37°C and heated at 70, 80, or 90°C for 15 min respectively. After the treatment, the anti-CD activities were determined and compared with the previous normal condition results.

### Antimicrobial Susceptibility Testing of BA PMC-80

Antibiotic susceptibility assay was performed to check whether BA PMC-80 carried any transmissible antibiotic resistance genes or not. The sensitivity of BA PMC-80 was assessed against a wide range of concentrations (200-0.02 g/ml) of methicillin, streptomycin, rifampicin, and vancomycin using the broth microdilution assay described in the earlier section.

### Toxicity Assessment of BA PMC-80

A subchronic toxicity test was performed on Syrian hamsters to evaluate the toxicity of BA PMC-80 following the slightly modified protocol previously used [44]. Briefly, 20 six-week-old, male Syrian hamsters, each weighing 100 g, were purchased from Central Lab Animal, Inc., Korea, and divided into 4 groups with five hamsters in each group. Hamsters were orally treated with a 1-mL daily dose of normal saline (group 1) and test bacteria (BA PMC-80) at concentrations of 1 × 10^8^ CFU/ml (group 2), 1 × 10^9^ CFU/ml (group 3) and 1 × 10^10^ CFU/ml (group 4) for 2 weeks. BA PMC-80 cells were prepared for the treatment from the overnight culture by centrifugation at 4,000 ×g for 10 min, and the bacterial pellet was then resuspended in PBS to make concentrations of 1 × 10^8^, 1×10^9^, and 1×10^10^ CFU/ml. Every day, the food consumption, health condition, and weight of the hamsters were recorded. All the animal experiments were done under the control of the Soonchunhayng Institutional Animal Care and Use Committee (SIACUC, Approval No. SCH19-0037).

### Evaluation of In Vivo Efficacy of BA PMC-80 in CDI Hamster Model

A CDI in vivo model was prepared using the Syrian hamsters to evaluate the efficacy of BA PMC-80 following a modified version of previous protocols (Fig. 7A) [45-47]. Briefly, 18 six-week-old male Syrian hamsters, each weighing 90-100 g, were purchased from Central Lab Animal, Inc., Korea, and divided into three groups with six hamsters in each group: 1) normal saline-treated infection control, 2) BA PMC-80 treated (1 × 10^10^ CFU/ml) and 3) vancomycin treated (2 mg/100 g) drug control. Hamsters were orally treated with 30 mg/kg clindamycin to disrupt the normal intestinal flora on day -2. Two days after the clindamycin treatment, on day 0, hamsters were orally infected with 1×10^4^ CFU/ml CD spores, previously prepared using the protocol [48] to initiate the infection. At 24 h post-infection, treatments of the hamsters were started with the feeding of normal saline, BA PMC-80, or vancomycin at day 1. Treatments were administered via oral gavage as a 1-ml single daily dose at the concentrations of 1×10^10^ CFU/ml (BA PMC-80) and 2 mg/100 g (vancomycin) for the next 14 days. Hamsters were observed throughout the experiment for signs of mortality and morbidity, the presence of diarrhea (wet tail), and overall appearance (activity, general response to handling, touch, ruffled fur). All the animal experiments were done under the control of the Soonchunhayng Institutional Animal Care and Use Committee (SIACUC, Approval No. SCH19-0037).

### Statistical Analysis

All experiments were done in triplicate. Statistical analyses were done using GraphPad Prism 9 software. Means of the drug-free control and test organism-treated group were compared using an unpaired Student's *t*-test (*p < 0.05, **p < 0.01, ***p < 0.001, ****p < 0.0001).

## Results

### Isolation and Screening of Bacteria with Anti-CD Activity

**Fig. 1:**
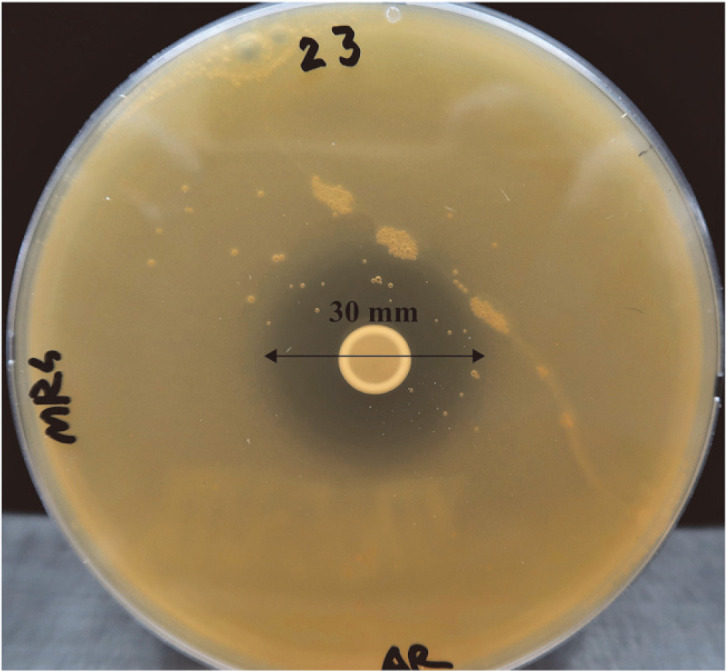
Anti-CD activity of BA PMC-80. Coculture assay showing BA PMC-80 bacterial spot inhibiting the growth of *C. difficile* by creating clear zone.

A total of 252 colonies were obtained and 49 types of colonies judged to be different based on morphology, color, and size were selected and stored at -80°C under 15% glycerol conditions. The selected strains were evaluated for anti-CD activity using an agar diffusion assay, and BA PMC-80 showed the most potent inhibitory activity against toxigenic *C. difficile* (ATCC 43255) with a 30 mm zone of inhibition (Fig. 1). Other strains showed various activities with a range of inhibition zones, including 0~5 mm, 5~10 mm, 10~15 mm, 15~20 mm, and 20~25 mm (not shown). Finally, it was decided to continue further studies with BA PMC-80.

### Characterization and Identification of BA PMC-80

**Fig. 2:**
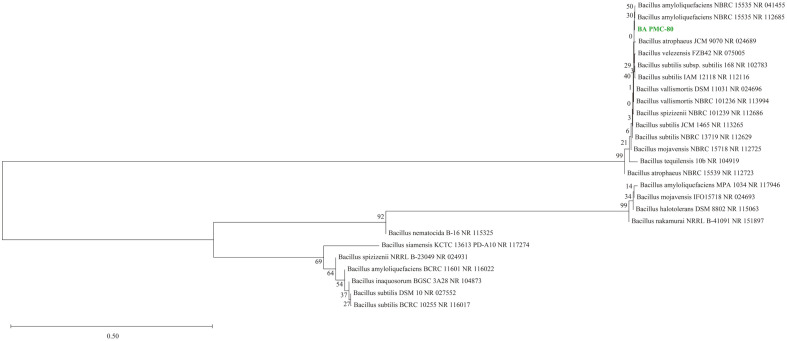
Phylogenetic tree of BA PMC-80 generated using neighbor-joining method. 16S rRNA gene sequence of the BA PMC-80 and similar strain from BLAST search are used in the tree construction. At major nodes, bootstrap percentages for 1,000 re-samplings are shown.

Based on the carbon source utilization profiles (Table 1), the API identification kit identified BA PMC-80 as *B. amyloliquefaciens* with 99.8% certainty. Over 99.0% similarity (Table 2) between the 16S rRNA gene sequence of BA PMC-80 and other *B. amyloliquefaciens* strains in the GenBank database further proved the identification. The constructed phylogenetic tree from the neighbor-joining method also demonstrated that BA PMC-80 was closely related to the *B. amyloliquefaciens* strains (GenBank, Accession No. NR_041455 and NR_112685) (Fig. 2). The identification results from API biochemical analysis, BLAST analysis, and phylogenetic analysis were consistent.

### Anti-CD Activity of BA PMC-80 CFS

**Fig. 3:**
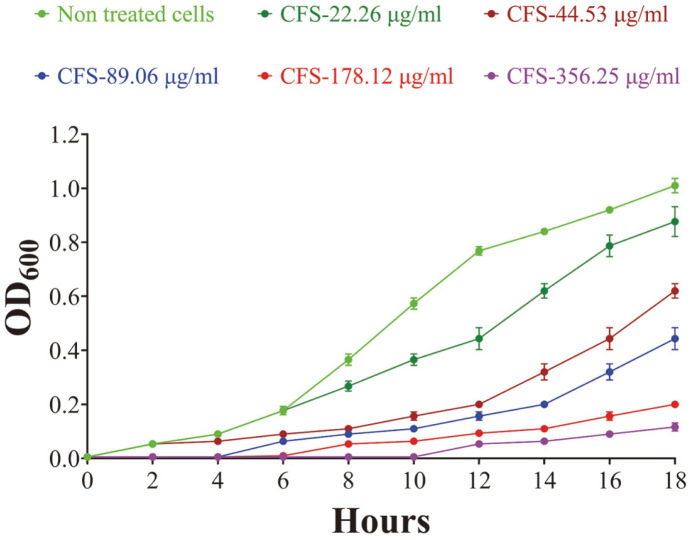
*C. difficile* growth inhibition by BA PMC-80. Growth was measured by broth microdilution assay using CFS of BA PMC-80 against *C. difficile*. The inhibition of *C. difficile* growth was confirmed by reducing the optical density measured at 600 nm. The navy-blue line represents the result of the CFS concentration of 89.06 μg/ml, which was found to reduce 50% OD compared to the positive control (lime-green line) and considered MIC_50_. The experiment was performed overnight (18 h) at 37°C in anaerobic conditions. All the broth microdilution experiments were performed in triplicate.

**Fig. 4:**
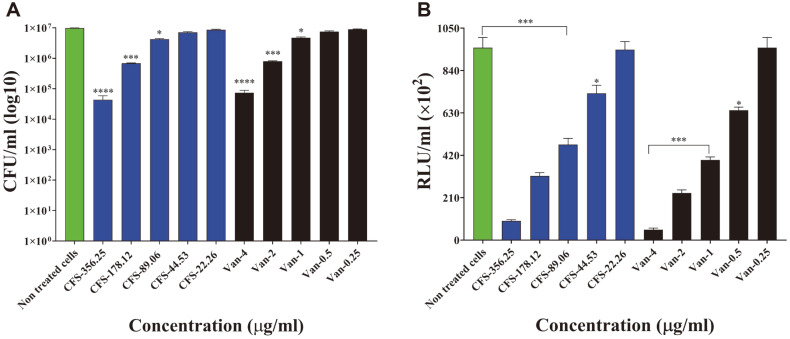
Antimicrobial activities of *B. amyloliquefaciens* PMC-80 (BA PMC-80) against *Clostridioides difficile* (CD ATCC 43255). (**A**) The dose-dependent reduction of colony-forming unit (CFU) in the CFU enumeration assay indicated the survival percentage of CD in CFS containing BHI broth media. (**B**) The dose-dependent decrease of ATP production in the cell viability assay verified the death of viable CD cells by BA PMC-80 CFS. ATP production is represented in relative luminescence unit/ml (RLU/ml). These experiments were carried out in triplicate. Data are given as mean values and standard deviations. *Statistical significance versus drug-free control using unpaired Student’s *t*-test (*p < 0.05, **p < 0.01, ***p < 0.001, ****p < 0.0001).

Along with the control drug vancomycin, BA PMC-80 CFS was used in a range of concentrations (5,700-2.78 μg/ml) to evaluate the anti-CD activity using broth microdilution assay. The CFS of BA PMC-80 exhibited a considerable anti-CD activity by reducing the OD in the broth microdilution assay with an MIC_50_ of 89.06 μg/ml (Fig. 3). Similar results were shown by the BA PMC-80 CFS in the CFU enumeration assay (Fig. 4A) and ATP measurement assay (Fig. 4B) with a consistent MIC_50_ value of 89.06 μg/ml.

### Stability of BA PMC-80 CFS under Different pHs, Temperatures, and Enzymatic Degradation

**Fig. 5:**
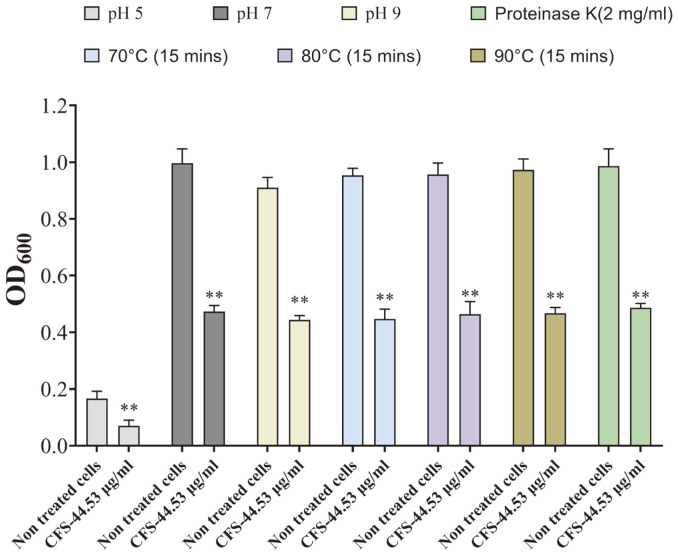
The stability of the anti-CD action of BAPMC-80 CFS under a range of adverse conditions. Concentration against *C. difficile* by optical density. The color of different bars represents different conditions like pH, heat, and Proteinase K treatment. All experiments were performed in triplicate and statistical analysis was performed in GraphPad prism software using student’s *t*-test (*p < 0.05) (**p < 0.01), (***p < 0.001).

The stability of the CFS of BA PMC-80 was evaluated by measuring its anti-CD activity in the presence of different pHs (5, 7, and 9), temperatures (70, 80, or 90°C) and Proteinase K (2 mg/ml) and by comparing the efficiency with the most active condition. BA PMC-80 CFS was found to be significantly effective against *C. difficile* in all three acidic (pH 5), neutral (pH 7), and basic conditions (Fig. 5). BA PMC-80 CFS also showed thermostability with considerable anti-CD activity after 15-min heat treatments up to 90°C (Fig. 5). Furthermore, we found that the Proteinase K treatment (2 mg/ml) did not influence the anti-CD activity of BA PMC-80 CFS (Fig. 5). Overall, the consistent 89.06 μg/ml MIC50 value in all the tested conditions confirmed that BA PMC-80 CFS contained considerably stable anti-CD compounds.

### Antimicrobial Susceptibility of BA PMC-80

**Fig. 6:**
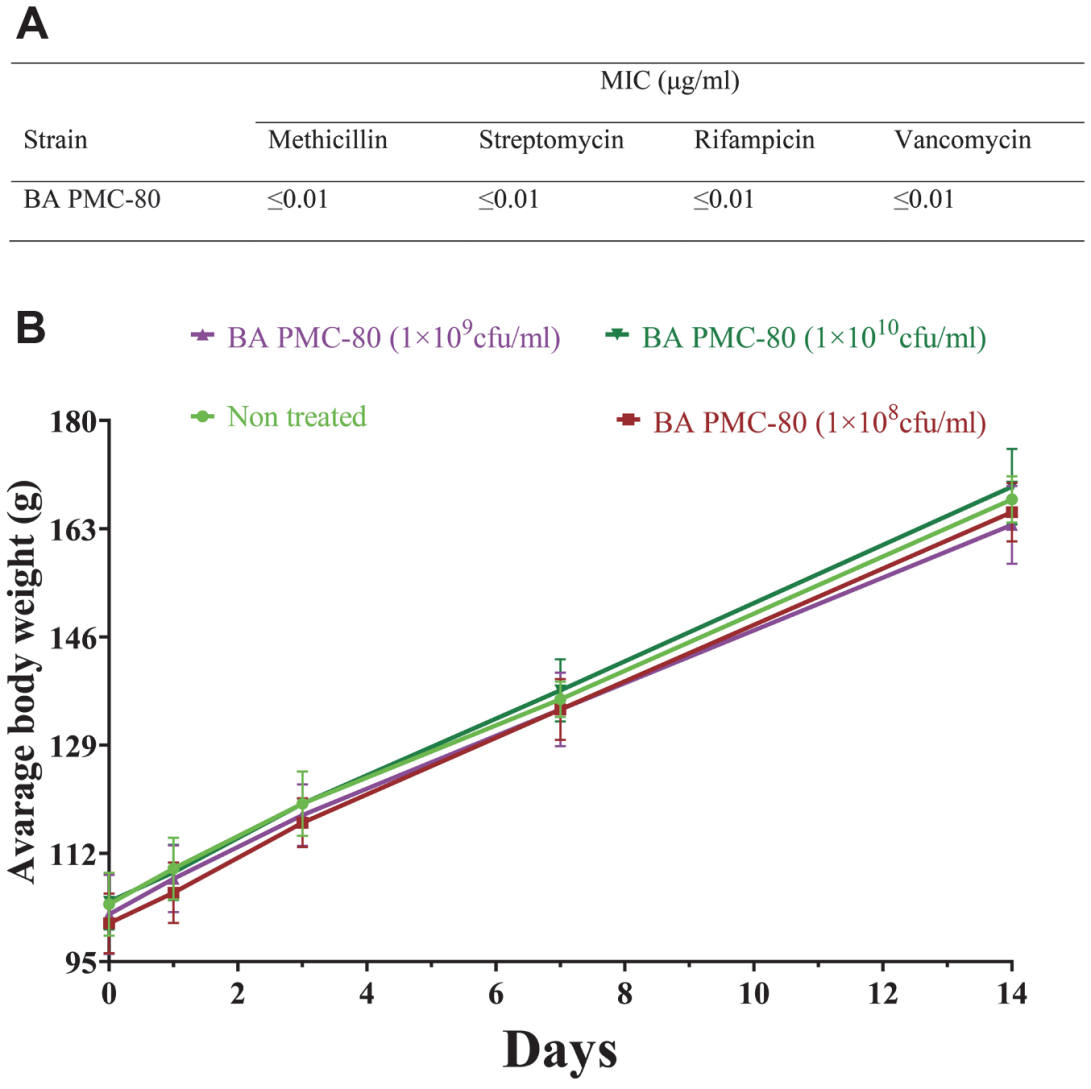
Safety assessment of BA PMC-80. (**A**) The result of antibiotic susceptibility test of BA PMC-80 against four antibiotics. (**B**) Line graph presents the weight gain of BA PMC-80-treated hamsters during the evaluation for subchronic toxicity.

BA PMC-80 did not contain any resistance genes to the tested antibiotics. The antibiotic susceptibility assay showed that BA PMC-80 was highly sensitive to all tested antibiotics (methicillin, streptomycin, rifampicin, and vancomycin) with the MICs of ≤ 0.01 μg/ml (Fig. 6A), and the results are comparable with the previous report [49].

### Toxicity of BA PMC-80

BA PMC-80 was non-toxic. The results from subchronic toxicity testing in hamsters showed no adverse effects, clinical symptoms, or death (Table S1) in any of the four test groups of hamsters after repeated 14-day oral feeding of BA PMC-80. However, a gradual increase in body weight was noticed in all the hamsters compared with their initial mean body weight (Fig. 6B). The food consumption and health status were almost the same (average 12-14 gm) during the experiment.

### In Vivo Anti-CD Effect of BA PMC-80 in Hamster CDI Model

**Fig. 7:**
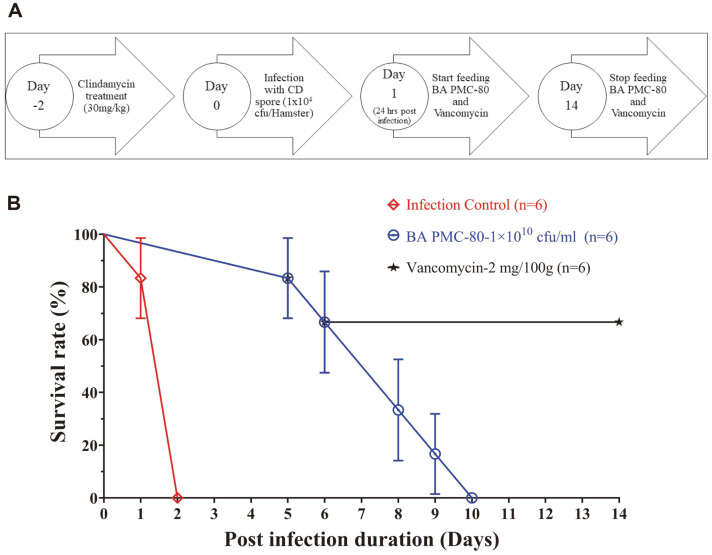
In vivo experimental design and outcomes. (**A**) The experimental design of the CDI hamster model shows the experiment was started with the exposure of clindamycin for the disruption of the intestinal normal flora, then infection with CD spore, and then treatment with BA PMC-80 or vancomycin until the completion of the study. (**B**) Survival rates of three groups of hamsters infected with *C. difficile* (ATCC 43255). The red line represents the normal saline-treated infection group, the navy blue line represents the BA PMC-80 group, and finally, the black line represents the vancomycin-treated group.

The Syrian hamster CDI model was used to evaluate the in vivo efficacy of BA PMC-80 against CDI along with the standard drug vancomycin. After completion of the experiment, it was found that all the normal saline-treated infection control hamsters expired by the end of day 2 (Fig. 7B). In contrast, the BA PMC-80 (1 × 10^10^ CFU/ml)-treated group showed a reduction in the infection severity and maintained a 100% survival rate until day 4 of the experiment. Later, the survival rate started to decrease with the death of hamsters, and finally, all the hamsters of this group expired on day 10, specifically, 1 out of 6 (16.66%), 2 out of 6 (33.33%), 4 out of 6 (66.66%), 5 out of 6 (83.33%), and 6 out of 6 (100%) hamsters on days 5, 6, 8, 9, and 10, respectively (Fig. 6B). Similar to BA PMC-80, in the control drug vancomycin (2 mg/100 g)-treated group, 1 out of 6 (16.66%), and 2 out of 6 (33.33%) hamsters expired on days 5 and 6. However, the remaining 66.66% survived till the end of the experiment (Fig. 6B).

## Discussion

*C. difficile* infection (CDI) is an increasing problem in hospitals, and the rising incidence of this disease has been associated with the overuse of broad-spectrum antibiotics and resistance to them [50]. Recently, the search for alternative treatments against CDI has gained extraordinary attention. Probiotics have already shown protective effects on CDI and have been included in many trials to recover the imbalance in the gut microbiota resulting from antibiotics [35, 51, 52]. This study provides information on the in vitro activity of a prospective probiotic strain *B. amyloliquefaciens* PMC-80 against *C. difficile* and its in vivo efficacy in a CDI hamster model.

BA PMC-80 was isolated from traditional fermented food and confirmed as *B. amyloliquefaciens* using a biochemical profile, 16S rRNA gene sequencing, and phylogenetic analysis. The results of our identification experiments are similar to those of several recent and previous articles [35, 39].

The inhibitory activity of BA PMC-80 against *C. difficile* in coculture experiment and the anti-CD activity of BA PMC-80 CFS with the MIC_50_ of 89.06 μg/ml in broth microdilution assay were significant. The results indicate that BA PMC-80 produces potent extracellular antimicrobial compounds against *C. difficile*, and it does not require cell-to-cell contact for the inhibition. Similar results were reported for the same and other probiotic bacteria against *C. difficile* in previous articles [35, 39, 42, 53]. The stable anti-CD activity of the CFS of BA PMC-80 under different pHs, temperatures, and Proteinase K matches the characteristics of the cyclic lipopeptides of *Bacillus subtilis* [29]. Other relevant studies also showed that *B. amyloliquefaciens* produces similar stable compounds such as difficidin, bacilysin, or LCI fusion protein to inhibit other bacterial pathogens such as *Erwinia amylovora* and *Ralstonia solanacearum* [31, 54]. So, our BA PMC-80 might have produced one of these antibiotic compounds against *C. difficile* in this study. However, further specialized characterization is required to confirm the exact anti-CD compound of BA PMC-80.

The sensitivity of BA PMC-80 against the four different contemporary antibiotics methicillin, streptomycin, rifampicin, and vancomycin indicates that it does not carry any resistance gene, and the result is consistent with the previous study [49]. Additionally, BA PMC-80 showed no subchronic toxicity in hamsters after 14 days of continuous oral treatment, and this result matches the result of a published article [55]. Both of these results suggest that BA PMC-80 has no side effects and is safe to consume.

Unlike the in vitro experiments, BA PMC-80 showed a mild effect in the in vivo hamster model. BA PMC-80 only prolonged the lifespan of the *C. difficile*-infected hamster by reducing the severity of the infection. It could not protect the treated hamster from death. A similar result was found by S. Geeraerts *et al*. in a broiler CDI model where *B. amyloliquefaciens* did not confer protection to the in vivo model but showed significant anti-CD activity in in vitro experiments [56]. However, several previous studies reported that *B. amyloliquefaciens* successfully conferred protection against C. Difficile-associated disease in a mouse model [57-59]. The difference in physiology and infection surface area between hamsters and mice might be the reason for the observed variation in *B. amyloliquefaciens* efficacy, as was previously proposed for auranofin [60].

In conclusion, BA PMC-80 showed a significant in vitro antimicrobial effect against *C. difficile* and a moderate in vivo effect in the hamster CDI model. Additional study is required to optimize the treatment conditions for the hamster CDI model and to discover the prime antimicrobial compound generated by BA PMC-80. To elucidate the protective mechanism of BA PMC-80, we will also need to evaluate its toxin-neutralizing activity, anti-inflammatory activity, and immunohistochemistry.

## Supplemental Materials

Supplementary data for this paper are available on-line only at http://jmb.or.kr.

## Figures and Tables

**Table 1:** The biochemical characteristics of *Bacillus amyloliquefaciens* PMC-80.

BA PMC-80			

Substrate	Reaction	Substrate	Reaction
Glycerol	+	Esculin ferric citrate	+
Erythritol	-	Salicin	+
D-Arabinose	-	D-Cellobiose	+
L-Arabinose	+	D-Maltose	+
D-Ribose	+	D-Lactose	-
D-Xylose	-	Melibiose	+
L-Xylose	-	D-Sucrose	+
D-Adonitol	-	D-Trehalose	+
Methyl-xylopyranoside	-	Inulin	+
D-Galactose	+	D-Melezitose	-
D-Glucose	-	D-Raffinose	+
D-Fructose	+	Amidon/ Starch	+
D-Mannose	-	Glycogen	+
D-Sorbose	+	Xylitol	-
Rhamnose	-	Gentiobiose	-
Dulcitol	-	D-Turanose	-
Inositol	-	D-Lyxose	-
D-Mannitol	+	D-Tagatose	+
D-Sorbitol	+	D-Fructose	-
α-Methyl-D-mannoside	-	L-Fructose	-
α-Methyl-D-glucoside	+	D-Arabitol	-
N-Acetylglucosamine	-	L-Arabitol	-
Amygdalin	+	Gluconate	-
Arbutin	+	2-Ketogluconate	-
		5-Ketogluconate	-

+, Positive; -, Negative

**Table 2:** Identification of isolated bacterial strain, BA PMC-80, based on 16S rRNA gene sequence analysis and their top ten close relatives published in NCBI databases.

NCBI reference	Organism	Length	Max score	Total score	Identities	E value
NR_117946.1	*Bacillus amyloliquefaciens* strain MPA 1034	1448	2606	2606	99.93	0.0
NR_075005.2	*Bacillus velezensis* strain FZB42	1550	2603	2603	99.79	0.0
NR_041455.1	*Bacillus amyloliquefaciens* strain NBRC 15535	1472	2603	2603	99.93	0.0
NR_024696.1	*Bacillus vallismortis* strain DSM 11031	1530	2603	2603	99.79	0.0
NR_112685.1	*Bacillus amyloliquefaciens* strain NBRC 15535	1475	2599	2599	99.86	0.0
NR_151897.1	*Bacillus nakamurai* strain NRRL B-41091	1508	2597	2597	99.72	0.0
NR_102783.2	*Bacillus subtilis* subsp. subtilis strain 168	1550	2597	2597	99.72	0.0
NR_116022.1	*Bacillus amyloliquefaciens* strain BCRC 11601	1468	2593	2593	99.86	0.0
NR_115325.1	*Bacillus nematocida* strain B-16	1511	2591	2591	99.65	0.0
NR_104873.1	*Bacillus subtilis* subsp. inaquosorum strain BGSC 3A28	1538	2591	2591	99.65	0.0
