# Supplementary material for: In Vitro and In Vivo Anti-Clostridioides difficile Effect of a Probiotic Bacillus amyloliquefaciens Strain
Source: J Microbiol Biotechnol. 2021 Oct 14;32(1):46–55. doi: 10.4014/jmb.2107.07057 (PMC9628829; doi:10.4014/jmb.2107.07057)
Supplement: Supplementary file 1 [file jmb-32-1-46-supple.pdf]

J. Microbiol. Biotechnol.
https://doi.org/10.4014/jmb.2107.07057

Table S1. Summary of fatality and clinical signs during the acute oral toxicity test.

Group	Dose (CFU/ml)	Fatality and clinical sign	Hours (Day 0) after dosing					Days after dosing													
			0.5	1	2	4	6	1	2	3	4	5	6	7	8	9	10	11	12	13	14
Group-1 (N=5)	0	Death	0	0	0	0	0	0	0	0	0	0	0	0	0	0	0	0	0	0	0
		NOA	5	5	5	5	5	5	5	5	5	5	5	5	5	5	5	5	5	5	5
		Mucous stool	0	0	0	0	0	0	0	0	0	0	0	0	0	0	0	0	0	0	0
		Irregular respiration	0	0	0	0	0	0	0	0	0	0	0	0	0	0	0	0	0	0	0
		Decrease in food intake	0	0	0	0	0	0	0	0	0	0	0	0	0	0	0	0	0	0	0
Group-2 (N=5)	1×10^8	Death	0	0	0	0	0	0	0	0	0	0	0	0	0	0	0	0	0	0	0
		NOA	5	5	5	5	5	5	5	5	5	5	5	5	5	5	5	5	5	5	5
		Mucous stool	0	0	0	0	0	0	0	0	0	0	0	0	0	0	0	0	0	0	0
		Irregular respiration	0	0	0	0	0	0	0	0	0	0	0	0	0	0	0	0	0	0	0
		Decrease in food intake	0	0	0	0	0	0	0	0	0	0	0	0	0	0	0	0	0	0	0
Group-3 (N=5)	1×10^9	Death	0	0	0	0	0	0	0	0	0	0	0	0	0	0	0	0	0	0	0
		NOA	5	5	4	4	4	3	5	5	5	5	5	5	5	5	5	5	5	5	5
		Mucous stool	0	0	0	1	1	2	0	0	0	0	0	0	0	0	0	0	0	0	0
		Irregular respiration	0	0	0	0	0	0	0	0	0	0	0	0	0	0	0	0	0	0	0
		Decrease in food intake	0	0	0	0	0	0	0	0	0	0	0	0	0	0	0	0	0	0	0
Group-4 (N=5)	1×10^{10}	Death	0	0	0	0	0	0	0	0	0	0	0	0	0	0	0	0	0	0	0
		NOA	5	5	5	5	5	5	5	5	5	5	5	5	5	5	5	5	5	5	5
		Mucous stool	0	0	0	0	0	0	0	0	0	0	0	0	0	0	0	0	0	0	0
		Irregular respiration	0	0	0	0	0	0	0	0	0	0	0	0	0	0	0	0	0	0	0
		Decrease in food intake	0	0	0	0	0	0	0	0	0	0	0	0	0	0	0	0	0	0	0

NOA, No observable abnormality
